# Diaphragm and Lung Ultrasonography During Weaning From Mechanical Ventilation in Critically Ill Patients

**DOI:** 10.7759/cureus.15057

**Published:** 2021-05-16

**Authors:** Funda Gok, Aysel Mercan, Alper Kilicaslan, Gamze Sarkilar, Alper Yosunkaya

**Affiliations:** 1 Department of Critical Care Medicine, Necmettin Erbakan University, Meram School of Medicine, Konya, TUR; 2 Department of Anesthesiology and Reanimation, Necmettin Erbakan University, Meram School of Medicine, Konya, TUR

**Keywords:** diaphragmatic ultrasound, lung ultrasound, weaning, critically ill patients, mechanical ventilation

## Abstract

Aim: Optimum timing is crucial to avoid negative outcomes of weaning. We aimed to investigate predictive values of diaphragmatic thickening fraction (DTF), diaphragmatic excursion (DE), and anterolateral lung ultrasound (LUS) scores in extubation success and compare with rapid shallow breathing index (RSBI) in patients extubated under traditional parameters.

Methods: Patients undergoing mechanical ventilation for >48 hours were included in the study. In patients planned for extubation, sonographic evaluations of the diaphragm and lung were performed at the T-tube stage. RSBI was achieved in the pressure support (PS) ventilation stage. Predictive values of DTF, DE, and anterolateral LUS scores were compared with RSBI in extubation success.

Results: Sixty-two patients were enrolled in the study. The study population consisted mostly of trauma patients (77%). A cut-off value of 64 was obtained for RSBI. The positive predictive value (PPV) was found at 97% in extubation success. Cut-off values of 27.5 for DTF, 1.3 cm for the DE, and 6.5 for LUS scores were obtained at the T-tube stage, respectively. PPVs of all sonographic parameters were found over 90%. At the first stage, weaning and extubation failures were determined as 35 and 9.6%, respectively. RSBI was found as a powerful parameter in determining extubation success (r=0.774, p≤0.001) and moderately correlated with sonographic parameters.

Conclusion: Investigating the lung and diaphragm via ultrasound provides real-time information to increase extubation success. Cut-off values of 64 for RSBI, 27.5 for DTF, 1.3 cm for the DE, and 6.5 for LUS scores were obtained, respectively, and PPVs of all sonographic parameters were found over 90%. We consider that sonographic evaluations accompanied by an RSBI will increase extubation success in the weaning process.

## Introduction

A great majority of the patients followed up in the intensive care units (ICU) are composed of those receiving mechanical ventilation (MV) therapy due to respiratory failure. Early termination or unnecessary delays in weaning leads to various negative consequences [1-3]. Among these results, the existence of serious consequences such as ventilator-associated pneumonia, ventilator-induced diaphragm atrophy, resumption of mechanical ventilator therapy indicates the importance of correct timing of weaning decisions.

The patient considered to be weaned is evaluated primarily under the clinical parameters. Besides, there are also some measurement parameters utilized by each clinician. The oldest and most widespread of such parameters is the rapid shallow breathing index (RSBI) [4]. However, since the breathing process is carried out by the diaphragm and auxiliary respiratory muscles, RSBI can be at desired values due to the auxiliary respiratory muscles, even if the diaphragm function is insufficient, meaning that RSBI values will be misleading in making the weaning decision [5,6].

Upon weaning, heart-lung interactions may lead to pulmonary edema increasing the burden of the heart. Such a condition is called weaning-induced pulmonary edema (WIPO) and is also a cause of weaning failure [7]. Additionally, other pathologies of the lungs such as pulmonary fibrosis, pulmonary hemorrhage, and diffuse pulmonary infiltrate are regarded to be among the causes of weaning failure [8]. 

In recent years, ultrasonography (US) has been used at the bedside by intensive care physicians in many areas such as the heart, lung, abdomen, kidneys, and vascular system. The assessment of the diaphragm and lungs through ultrasonography at the weaning stage has drawn attention [9]. In studies where the diaphragm was interpreted sonographically, diaphragmatic thickening fraction (DTF) and diaphragmatic excursion (DE) were measured [5,6,10-13]. However, lung ultrasound (LUS) enables the loss of lung ventilation induced by WIPO and other mechanisms to be evaluated [7]. In the examination, aeration changes in the lung are scored (LUS score) [14,15]. Although the studies rating the diaphragm sonographically during the weaning are also sufficient, the number of studies where the diaphragm and lung are sonographically investigated concurrently is limited [9,11,12]. This study, it was aimed to determine the predictive values of DTF, DE, and LUS scores in extubation success by evaluating the diaphragm and lung sonographically in patients extubated under conventional parameters and to compare them with RSBI.

## Materials and methods

Study population

After obtaining the approval of the local ethics committee and informed written consent from the patients’ next of kin, this prospective, observational study was conducted. Patients (18-80 years old) who received MV (>48 hours) in the ICU of the Meram Medical School Hospital, between October 2017 and September 2019 were included in the study. Patients with neuromuscular disease, diaphragmatic dysfunction, presence of thoracostomy, pneumothorax, pneumomediastinum, obesity (body mass index (BMI) 30 ≥ m^2^/kg), chronic obstructive pulmonary disease, pregnancy, and known heart failure were excluded from the study. Patients undergoing MV support were evaluated under the traditional weaning criteria (FiO_2_<0.5, PEEP ≤ 5, PaO_2_/FiO_2_ >200, breath counts <30/minute, elimination or regression of the need for MV, the existence of consciousness or cough reflex in the patient, the patient’s hemodynamic stability, the stability of metabolic functions) by the primary physician. Spontaneous breathing trials (SBT) were performed in patients meeting the above-mentioned criteria.

The Stage of Pressure Support Ventilation

In the first stage, the patient was switched to pressure support (PS) ventilation mode [continuous positive airway pressure (CPAP): Drager Evita XL]. While the values of PS and positive and expiratory pressure (PEEP) were 5 cmH_2_O and 5 cmH_2_O, respectively. RSBI was achieved in the PS ventilation stage.

The T-Tube Stage

If no negative changes described below were observed in the patient, the T-tube stage was initiated as the second stage. Oxygen support was administered from the T-tube to achieve a peripheral oxygen saturation of ≥92. The sonographic measurements of the patient with the T-tube were performed, and the patient was extubated when the clinician decided.

Weaning failure was defined as the patient’s failure in SBT (PS ventilation or T-tube stage) or extubation failure. Such features as changes in mental status during PS ventilation or T-tube, stages sweating, respiratory rate of >37 breaths per minute, hemodynamic instability (heart rate >140, systolic blood pressure ≥ 180 mm/hg and <90 mm/hg), and an increase in respiratory workload were considered SBT failure. If the patients were re-intubated within 48 hours, or if noninvasive mechanical ventilation (NIMV) was carried out, the extubation was defined as extubation failure [8]. The study aims to obtain a cut-off value for DTF, DE, and LUS scores through the sonographic evaluation of the lung and diaphragm in the patients for whom the weaning process developed successfully and those undergoing extubation after the first weaning trial and to evaluate the correlation between those values and RSBI.

The sonographic evaluations were performed by the same physician experienced in the field at the stage T-tube after the patients completed approximately a 30-minutes to 1-hour period. The physician evaluating those outcomes was ineffective in decision-making to the patients’ weaning.

Diaphragmatic Ultrasonography

**Figure 1:**
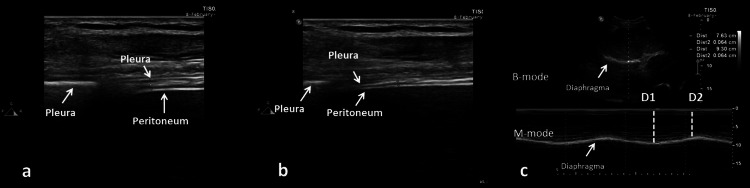
Diaphragmatic thickness after the inspiration (a), expiration (b), diaphragmatic excursion (c) Diaphragmatic thickness was obtained by examining the right diaphragm with a linear probe. The hypoechoic muscle layer located between the pleura and peritoneum is the diaphragm. DE taken in brightness (B) and motion (M) modes (c). The image was obtained via the examination of the right diaphragm using a convex US probe. The diaphragm is monitored as a hyperechoic line (white arrow) around the liver. DE during the inspiration (distance, D1) and expiration (distance, D2) can be calculated based on D1–D2. In this image, DE was found as 9.30−7.73=1.67 cm. DE: diaphragmatic excursion, US: ultrasound.

**Figure 2:**
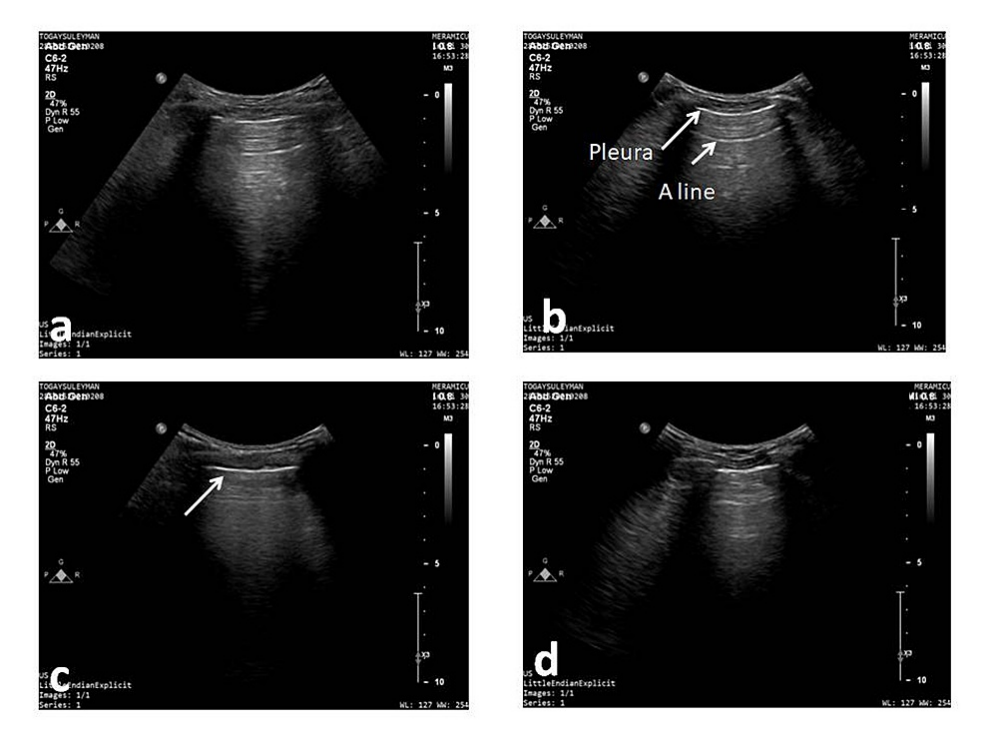
Lung ultrasonography The lateral upper (a), lower (b), anterior upper (c), and lower (d) sonographic images of the lung. In images (a), (b), and (d), A-line was obtained in and below the pleura (LUS score=0). However, only the pleura is seen in image (c). B-lines are prominent in the dynamic image (LUS score=2). LUS: lung ultrasonography.

The sonographic evaluations were performed as described in previous studies investigating the same entity [5,6,13]. The measurements of DTF were performed in the zone of apposition through a linear probe (L12-4 MHz, Philips Affiniti, Philips, Amsterdam, Netherlands). The diaphragm thickness was measured at the end of inspiration and expiration (Figure 1). DTF was calculated with the following formula: DTF = end-inspiration diaphragm thickness − end-expiration diaphragm thickness/end-expiration diaphragm thickness × 100. The convex probe (C4-2 MHz, Philips Affiniti 30) was used to obtain DE. The probe was placed in the intercostal space, and the displacement of the diaphragm was measured in motion (M) mode (Figure 2). For DTF and DE measurements, five separate measurements were performed, and the average scores were calculated.

Lung Ultrasonography

The examinations were carried out with a convex probe. By using the anterior axillary line as the landmark, the lung was first divided into anterior and lateral regions, and then each region was divided into upper and lower areas. A total of eight areas, four areas for each hemithorax, were evaluated sonographically. The following scoring criterion was used for the evaluation (Score 0: A-lines, lung sliding, two or fewer than B-lines, Score 1: three or more than B-lines, Score 2: multiple coalescences of B-lines, Score 3: pulmonary consolidation or presence of tissue pattern) [16]. The total of the scores from the anterior and lateral regions was recorded as the anterolateral score (ranging from 0 to 16; Figure 2).

## Results

**Figure 3:**
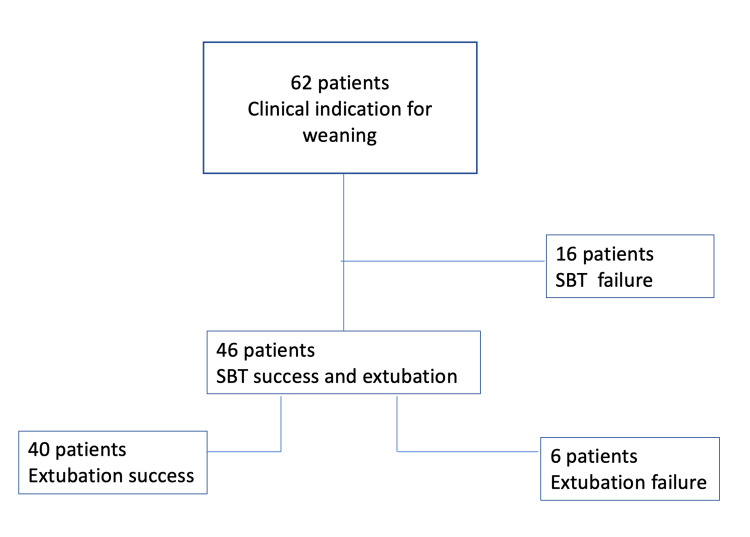
Weaning outcomes Forty-six patients completed the stages of SBT successfully and were extubated. Sixteen (25.8%) patients could not be extubated at the first attempt. Among the extubated patients, six (9.6%) were defined as extubation failure. While five patients were re-intubated within 48 hours, one patient underwent NIMV within 48 hours. A total of 22 (35%) patients were defined as weaning failure. SBT: spontaneous breathing trial, NIMV: noninvasive mechanical ventilation.

The study population (n=62) was hospitalized due to trauma (77%), sepsis (16%), intoxication (3%), and postoperative hemorrhage (3%). Forty-six patients completed the stages of SBT successfully and were extubated. Sixteen (25.8%) patients could not be extubated at the first attempt. Among the extubated patients, six (9.6%) were defined as extubation failure. While five patients were re-intubated within 48 hours, one patient underwent NIMV within 48 hours (Figure 3). The reason why those were re-intubated was evaluated as tracheobronchitis and mucous plugging. A total of 22 (35%) patients were defined as weaning failure.

There was no difference between the Acute Physiology and Chronic Health Evaluation-2 (APACHE-2) scores in patients. In patients with successful extubation, while the number of intubation days and the duration of ICU and hospital stay were shorter, the mortality rate was seen to be lower, compared with the other patients. RSBI score was also lower in patients with successful extubation than that in other patients (Table 1).

**Table 1:** Demographic and clinical characteristics BMI: body mass index, APACHE: Acute Physiology and Chronic Health Evaluation, RSBI: rapid shallow breathing index, ICU: intensive care unit, SBT: spontaneous breathing trial, PS: pressure support. ^#^In patients with successful extubation, while the number of intubation days, the duration of ICU, and hospital stay were shorter compared with the other patients.

	All (62)	Extubation Success (40)	SBT Failure (16) (PS &T-tube)	Extubation Failure (6)	p-Value
Age	57.6 ±14.1	58.7 ± 14.3	56.3 ± 15.2	59.5 ± 10.2	0.45
Male/female (n)	42/20	27/13	11/5	4/2	
BMI	25.8 ± 2.7	25.8 ± 2.6	25.5 ± 3.8	27.5 ± 1.6	0.21
APACHE II	12.2 ± 4.2	12.9 ± 3.5	13.6 ± 2.9	12.8 ±4.5	0.48
Duration of endotracheal intubations (day)	10.3 ± 4.6	9.6 ± 3.3	15.6 ± 2.5	12.5 ± 4.4	≤0.001^#^
ICU length of stay	18.3 ± 8.6	14.7 ± 2.7	22.1 ± 3.4	21.8 ± 4.3	≤0.001^#^
Hospital length of stay	25.7 ± 11.1	21.3 ± 8.8	28.5 ± 9.2	30.8 ± 16.7	≤0.001^#^
Mortality (n/%)	8 (12)	2 (5)	4 (25)	2 (33)	

RSBI was achieved in the PS ventilation stage. Patients were evaluated sonographically at the T-tube stage. A cut-off value was determined for the variables of RSBI, DTF, DE, and LUS scores (Table 2).

**Table 2:** RSBI and sonographic parameters for extubated patients (n=46) RSBI: rapid shallow breathing index, DTF: diaphragm thickening fraction, DE: diaphragmatic excursion, LUS: lung ultrasound, ROC: receiver operating characteristic, AUC: area under the curve, PPV: positive predictive value, NPV: negative predictive value.

	Cut off	ROC of AUC	Sensitivity (%)	Specificity (%)	PPV (%)	NPV (%)	95%CI
RSBI	64	0.908	82.5	83.3	97	0.4	0.817–0.970
DE	1.31	0.792	69	67.5	96	27.8	0.586–0.997
TDF%	27.5	0.808	67.5	66.6	95	24	0.650–0.967
LUS score	6.5	0.760	70.0	83.3	96	26	0.531–0.990

A correlation was detected between extubation success and various parameters. Therefore, while there was a strong correlation between extubation success and RSBI (r=0.774, p≤0.001), a moderate correlation was detected between extubation success and all sonographic parameters. The correlation coefficients were found as DE (r=0.532, p≤0.001), DTF (r=0.499, p≤0.001), and LUS score (r=0.396, p≤0.05).

## Discussion

In the present study, the sonographic evaluation of the diaphragm and lung was performed at the T-tube stage in the patients extubated under the traditional parameters in ICU. The sonographic values obtained at T-tube stages had a positive predictive value (PPV) over 90% in determining the success of extubation. It was found out that RSBI is a very powerful parameter in determining extubation success and correlated with sonographic parameters.

RSBI value defined for successful weaning is 100-105 breaths/minutes/liter [4]. Lower predictive values have been reported in different studies [10,17,18]. Patient population or methodological differences cause a wide range of results for RSBI. In our study, the cut-off value was detected as 64 for RSBI and assessed to be compatible with that obtained in many studies [10,17].

In the evaluation of diaphragmatic function, it has been reported that DTF is of a better performance than RSBI and DE; the measurements of diaphragm thickening are more reliable or accurate to evaluate diaphragmatic function in those supported by MV therapy [5,10,19,20]. In studies conducted so far, the cut-off values of DTF for weaning vary between 20% and 36% [5,10-12,21,22]. The methodological differences used in the studies may have led to different cut-off values. In our study, however, the majority of study participants were composed of trauma patients, and if the first weaning was failed, the later stages were not evaluated sonographically. In one study where the sonographic examinations were performed at different PS levels (PS 5, 10, and 15 cmH_2_O), the cut-off value for DTF was reported as 20%. Based on the study findings, as PS decreased, the predictivity of DTF for extubation was found to increase [22]. In a study, DiNino et al. [5] performed sonographic evaluations in some patients during PS (5 cmH_2_O) and in others during the inhalation with T-tube and reported that DTF estimates the extubation success equally through the evaluations of PS and T-tube. Our results are compatible with the findings of the study by DiNino et al. [5] predicting the extubation success of DTF as ≥30 with a PPV of 91%.

It is known that DE is positively correlated with inspiratory volumes [23]. Given the amplitude of diaphragmatic movements, the excursion values of ≤10 mm are associated with diaphragm dysfunction and prolonged MV [6]. In our study, the cut-off value of DE was found as 1.31 cm during the T-tube stage, and to have PPVs of 93% and 96% in determining the success of extubation, respectively.

All sonographic evaluations were conducted by the same physician experienced in the field. The difficulty of sonographic measurements was not evaluated with an objective scale; however, the evaluation of diaphragm thickness is considered to be more difficult, compared with DE. The diaphragm is located between the pleura and the peritoneum and is the muscular layer appearing to be hypoechoic sonographically. It is difficult to evaluate in obese patients or patients with thick skin and subcutaneous tissue due to such factors as edema or hematoma. In lean patients, however, intercostal muscles located above the diaphragm prevent a clear assessment of the diaphragmatic image. Such a measurement is difficult, especially when the patient experiences tachypnea during SBT. In the study by Whebell et al. [24], the diaphragmatic thickness was reported not to be evaluated in four patients due to increased BMI. Even so, it is easy to evaluate DE sonographically. However, such characteristics as the impedance of adjacent structures, abdominal compliance, PEEP, and ventilation support are known as the factors which may affect the measurement of DE [5,19,20]. In a study, the diagnostic accuracy of global and regional LUS scores in ARDS was compared in critically ill patients undergoing MV [25]. While the researchers evaluated the anterior, lateral, and posterior areas as regional LUS, the score obtained from all areas was calculated as the global LUS score [25]. In another study examining 12 regions, weaning was reported to be successful if the LUS score was <12 [12]. Posterior regions have higher diagnostic accuracy in the assessment of hydrostatic pulmonary edema due to atelectasis and infiltration [26-28]. However, compared to the posterior score with the global score, the diagnostic accuracy of the posterior score is low in ARDS patients, and the removal of the posterior regions from the global score leads to no change in the diagnostic accuracy [25]. The anterolateral score was concluded to display as a good performance as the global score in the diagnosis of ARDS [25]. Additionally, considering ICU patients, many factors, such as catheters, cables, and patients’ positioning make the posterior examination difficult. In our study, only anterolateral lung regions were evaluated by taking the aforementioned reasons into account. Increased LUS score is an independent risk factor for weaning failure in the elderly [14]. In the study by Bouhemad et al. [14], the condition has been reported to be associated with extubation failure if the anterolateral LUS score is ≥5. In our study, the cut-off value found for the LUS score was proximate to that found by Bouhemad et al. For the success of extubation, the cut-off value of 6.5 was obtained during the T-tube stage.

The weaning process is affected by many factors from consciousness and cardiac functions to the patients’ physiological status, as well as the length of MV therapy administered to the patients [8]. When the patient is put into SBT, the failure is related to either cardiovascular dysfunction or the inability of the breathing pump to support the respiratory load [8]. Based on the literature, weaning failure has been defined as the patient's inability to wean from MV or re-intubation following the extubation as a result of SBTs [18,29]. In our study, 16 patients were not successful after the first attempts of weaning. Although the support of MV was continued for a while in these patients, the outcomes were not re-evaluated for the study. A significant increase was witnessed in terms of LUS score in two patients. Since weaning failure was considered to originate from cardiac reasons, only these two patients underwent advanced cardiac evaluation. In those extubated albeit successful SBT, the rate of re-intubation within 48 hours varies between 13% and 26% [12,30]. In our study, as consistent with the findings reported in those results, the rate of extubation failure was detected as 10%. It has been determined the reasons for re-intubation as tracheobronchitis and lack of sufficient cough reflex and, the weaning failure rate as 35%. The rate of weaning failure after a single attempt was between 26% and 42% and our study findings are compatible with those stated in the studies [8,18]. The studies conducted so far are those in which the sonographic evaluations have been performed in the patients considered to be weaned under the clinical evaluation and mechanical parameters. In the future, the studies in which weaning will be decided with all the cut-off values obtained will further reveal the importance of sonography in the field. Therefore, it is essential that an international standard criterion, including also echocardiographic evaluations, should be determined to elucidate the weaning process. In our study, no echocardiographic investigation was performed in the patients with weaning failure, and such an outcome is a limitation of our study.

## Conclusions

In conclusion, the examination of the lung and diaphragm through ultrasonography during the weaning process provides both morphological and functional real-time information. Cut-off values of 64 for RSBI, 27.5 for DTF, 1.3 cm for a DE, and 6.5 for LUS scores score were obtained, respectively, and PPVs of all sonographic parameters were found over 90%. We consider that the sonographic examinations accompanied by RSBI should be performed to increase the success of extubation.
